# Regulatory effects of plant growth regulators and micro-fertilizers on recovery of fragrant rice seedlings from simulated transplanting injury

**DOI:** 10.3389/fpls.2026.1770799

**Published:** 2026-02-04

**Authors:** Xiangyi Yu, Xiaoqing Du, Xiaoying Liang, Zixuan Wang, Zhaowen Mo

**Affiliations:** College of Agriculture, South China Agricultural University, Guangzhou, China

**Keywords:** antioxidant enzymes, fragrant rice seedling, growth, micro-fertilizers, plant growth regulators, transplanting injury

## Abstract

Plant growth regulators (PGRs) and micro-fertilizers (MFs) and are widely used to modulate crop growth and physiological processes. This study evaluated the foliar application of specific PGRs and MFs on promoting fragrant rice seedling recovery after simulated transplanting injury. Two fragrant rice cultivars (Xiangyaxiangzhan and Yuxiangyouzhan) were grown hydroponically and exposed to an artificial treatment to simulate mechanical transplanting injury. The experiment comprised four treatment groups, foliar-sprayed of 100 mg·L^-1^ gibberellin (T1), 2 mg·L^-1^ indolebutyric acid (T2), Mg fertilizers (T3, 2 mg·L^-1^ Mg^2+^), and Zn fertilizers (T4, 2 mg·L^-1^ Zn^2+^), respectively. We established two control groups, including a non-injured (CK0) and a simulated root injury (CK) treatment, and neither received PGRs or MFs. The seedling growth morphology and physiological indices were measured and analyzed. The results demonstrated that PGR and MF application significantly altered the growth and physiological performance of rice seedlings under simulated transplanting injury, with effects varying by treatments and varieties. Compared with CK, the T1 treatment increased stem fresh weight (25.88-38.4%), stem fresh weight ratio, plant height, stem and leaf length, leaf catalase activity, stem superoxide dismutase activity, and reduced leaf malondialdehyde content. The T2 treatment induced changes in antioxidant enzyme activities, specifically leading to an enhancement in stem superoxide dismutase activity (112.15-165.64%). The T3 treatment significantly increased the root-shoot ratio (21.74-23.07%), root fresh weight ratio, root dry weight ratio, and superoxide dismutase activity in root and stem relative to CK. The T4 treatment significantly increased the root fresh weight ratio (18.32-43.03%), superoxide dismutase activity in various tissues, and peroxidase activity in root relative to CK. These findings indicate that PGRs and MFs can effectively regulate growth and antioxidant response in fragrant rice seedlings under simulated mechanical transplanting injury.

## 1 Introduction

Rice is a major food crop, serving as the staple food for more than half of the global population (Danso Ofori et al., 2025). With the continual growth of the world’s population, a further increase in total rice yield will be necessary over the next decade (Subudhi, 2023). Driven by a shortage of labor, the mechanization of rice cultivation has become an essential development for China, a major produce (Chen et al., 2018; Tao et al., 2019; Min et al., 2021).

Mechanized rice farming primarily employs two methods: mechanical transplanting and direct seeding (Hong et al., 2022). Despite its wider adoption owing to stable yields from controlled nursery conditions, mechanical transplanting has the fundamental drawback of unavoidable transplanting injury (Xing et al., 2017; Yu et al., 2019). Transplanting injury adversely affects seedlings by delaying rejuvenation and hindering establishment, which negatively impacts final yield and quality formation (Singh and Singh, 2014). Researchers have reported that mechanical transplanting results in a 31.26% lower rice grain yield than manual transplanting (Liu et al., 2015). Therefore, alleviating transplanting injury in mechanical rice transplanting is of paramount importance for achieving high yields and superior quality.

Traditional nursery-based cultivation enhances transplanted rice yield primarily by boosting seedling vigor through improved dry matter and nutrient content (Ros et al., 2003). This effect can be achieved through the foliar application of PGRs and MFs during the rice seedling stage. Previous studies on PGRs, such as the use of N-acetylcysteine priming to boost antioxidant and photosynthetic performance (He et al., 2022), have shown efficacy in mitigating mechanical transplanting injury. Similarly, MFs applied via foliar spray hold potential for comparable benefits. Orysastrobin mitigates transplanting injury in rice through blocking transpiration, maintaining water content, and scavenging ROS, which curbs hydrogen peroxide overproduction and water loss (Takahashi et al., 2017). Soaking seeds with 50 mg L^-1^ melatonin improved root regeneration, cell division, elongation, and chlorophyll content, which in turn facilitated seedling recovery and establishment following mechanical transplanting (Lin X, et al., 2018). Root soaking with brassinolide repairs transplanting injury in machine-transplanted rice seedlings by elevating antioxidant enzyme activities and endogenous hormone levels, thereby reducing hydrogen peroxide content. (Li et al., 2020a). Triacontanol alleviates transplanting injury in mechanically transplanted rice by enhancing the seedling antioxidant system and modulating key enzymes in the ASA-GSH cycle, thereby improving the ASA/GSH redox state, maintaining photosynthetic capacity (Li et al., 2016). Micro-fertilizers (MFs) have been shown to exert effects equivalent to those of PGRs. For example, optimal selenium supply promotes rice seedling growth by enhancing antioxidant capacity (Liu et al., 2024). Elevated Fe²^+^ from rice straw return paradoxically enhances iron uptake and reduces root oxidative stress yet ultimately inhibits rice seedling growth (Li et al., 2025). Therefore, PGRs and MFs are crucial for mitigating the injury caused to rice seedlings by mechanical transplanting.

Previous studies have demonstrated that plant growth regulators (PGRs), such as indolebutyric acid and gibberellin, and micro-fertilizers (MFs), including Mg and Zn fertilizers, can enhance shoot and root growth in plants (Chou et al., 2011; Prom-u-thai et al., 2012; Stuepp et al., 2015; Matusmoto et al., 2016; Singh Patel et al., 2022). Based on these findings, it was hypothesized that PGRs and MFs may also regulate the recovery of rice seedlings from transplanting injury. The present study thus aimed to investigate the regulatory effects of micro-fertilizers and plant growth regulators on the recovery of rice seedlings under simulated transplanting injury conditions. A hydroponic experiment was conducted using two fragrant rice cultivars subjected to artificially simulated mechanical transplanting injury. The plants were grown under four different PGR and MF treatments, and seedling growth morphological traits along with physiological parameters were measured to evaluate their regulatory effects on transplanting injury alleviation.

## 2 Materials and methods

### 2.1 Experiment description and treatments

This experiment was conducted at the College of Agriculture, South China Agricultural University (SCAU), Guangzhou, China. The fragrant rice varieties selected for the experiment were the indica cultivars Xiangyaxiangzhan and Yuxiangyouzhan, which were provided by the College of Agriculture, SCAU. Xiangyaxiangzhan and Yuxiangyouzhan are popular high-quality fragrant rice varieties in South China. Xiangyaxiangzhan is a conventional indica rice variety characterized by poor agronomic performance and environmental susceptibility (Liang et al., 2023), but also by its superior aroma and flavor quality (Qing et al., 2022). Yuxiangyouzhan is a super rice variety notable for its high productivity and wide adaptability (Chen et al., 2023). It exhibits vigorous growth and strong resistance to environmental stresses (Liu et al., 2020), and has shown particularly strong performance under low-temperature conditions (Li et al., 2019). Given the known differences between the two fragrant rice varieties, Xiangyaxiangzhan and Yuxiangyouzhan were selected as experimental materials to investigate the effects of the PGRs or MFs on recovery of fragrant rice seedlings from simulated transplanting injury.

The experiment included four treatment groups: T1, foliar-sprayed with 100 mg·L^−1^ gibberellin; T2, foliar-sprayed with 2 mg·L^−1^ indolebutyric acid; T3, treated with Mg fertilizer (2 mg·L^−1^ Mg²^+^); and T4, treated with Zn fertilizer (2 mg·L^−1^ Zn²^+^). Two control groups were also established: a non-injured control (CK0) and a simulated root-injured control (CK), neither of which received PGRs or MFs. Rice seeds were soaked, germinated, and raised in a nursery. Uniform seedlings at the one-leaf stage were selected and cultivated hydroponically for 14 days. The hydroponic pots measured 65.0 cm (L) × 41.0 cm (W) × 15.5 cm (H). Seedlings were secured using floating boards with uniformly spaced circular holes (2.0 cm diameter, 4.0 cm center-to-center distance). The experiment consisted of six hydroponic pots, each containing seedlings of both cultivars, Xiangyaxiangzhan and Yuxiangyouzhan. On the 8^th^ day of hydroponic cultivation, seedlings underwent a simulated root injury treatment, wherein roots were trimmed to a uniform length of 1.5 cm to mimic the damage associated with mechanical transplanting. Foliar sprays were then applied daily for three consecutive days following the injury. On the 14^th^ day, seedlings were sampled for the measurement of growth, morphological, and physiological indices.

The nutrient solution for the seedling growth in the pot was the Kimura B nutrient solution and was prepared according to Huang et al. (2020), and the pH value was adjusted to 4.7–4.9. The nutrient solution included the following: Stock 1 of 500 mL with KNO_3_ and Ca(NO_3_)_2_·4H_2_O; Stock 2 of 500 mL with MgSO_4_·7H_2_O, K_2_SO_4_, and (NH_4_)_2_SO_4_; Stock 3 of 500 mL with KH_2_PO_4_; Stock 4 of 1000 mL with FeSO_4_·7H_2_O and Na_2_EDTA; and Stock 5 of 500 mL with MnCl_2_·4H_2_O, ZnSO_4_·7H_2_O, CuSO_4_·5H_2_O, H_3_BO_3_, and (NH_4_)_6_Mo_7_O_24_·4H_2_O (Zhuang et al., 2025). The nutrient solution was replaced every three days.

### 2.2 Sampling and measurement

#### 2.2.1 Morphological parameter measurements

Morphological measurements were conducted on uniformly grown, representative seedlings from each treatment. Randomly sampled plants were used to measure plant height, root length, stem length, leaf length, leaf width, number of green leaves per plant. For biomass determination, plants were separated into root, stem, and leaf tissues. Fresh weights were immediately measured (analytical balance, PRACTUM124-1CN). Tissues were then dried at 80°C until constant weight before dry weight measurement. Measurements were repeated four times, with ten plants per replicate. The distribution of biomass among different plant parts was then calculated. The specific leaf weight was calculated using formula: leaf weight/leaf area. The root-shoot ratio was calculated using the formula: dry weight of belowground parts/dry weight of aboveground parts.

#### 2.2.2 Physiological parameter measurements

The root, stem, and leaf samples from representative seedlings were collected for physiological assays, frozen immediately in liquid nitrogen, and stored at -80°C for subsequent analysis. The activities of antioxidant enzymes, including catalase (CAT), superoxide dismutase (SOD), and peroxidase (POD), as well as the malondialdehyde (MDA) content, were determined following the method of Li et al. (2019) with minor modifications. Briefly, crude enzyme extracts were prepared by homogenizing 0.1 g of fresh tissue in phosphate buffered saline (PBS, pH 7.8), followed by centrifugation at 8,000 rpm for 15 min at 4°C (Drummond and Austin-Tse, 2013).

SOD activity was assayed by adding the extract to a reaction mixture containing sodium phosphate buffer, methionine, nitroblue tetrazolium chloride, and EDTA. After adding riboflavin, the mixture was exposed to 4000 lx light for 20 min, and absorbance was read at 560 nm. One unit of SOD was defined as the amount causing 50% inhibition of the control reaction (Li et al., 2020b). POD activity was determined by mixing the extract with PBS and guaiacol, initiating the reaction with hydrogen peroxide, and monitoring the absorbance change at 470 nm every 30 s for 2.5 min (Lin et al., 2024). CAT activity was measured by adding the extract to a mixture of ultrapure water and hydrogen peroxide. The decrease in absorbance at 240 nm was recorded every 30 s for 2 min. One unit was defined as a decrease of 0.01 Abs per minute (Pan et al., 2013). MDA content was analyzed by heating the extract with thiobarbituric acid in a boiling water bath for 30 min. After cooling and centrifugation, absorbance of the supernatant was measured at 532, 600, and 450 nm for calculation (Li et al., 2020b).

### 2.3 Data analysis

Experimental data were recorded and organized using Microsoft Office 2013, and means and standard deviations (SE) were calculated. Analysis of variance and multiple comparisons using the Least Significant Difference (LSD) method were performed using Statistix 8.0. Results were considered significant when p < 0.05.

## 3 Results

### 3.1 Total biomass and growth parameters

**Figure 1:**
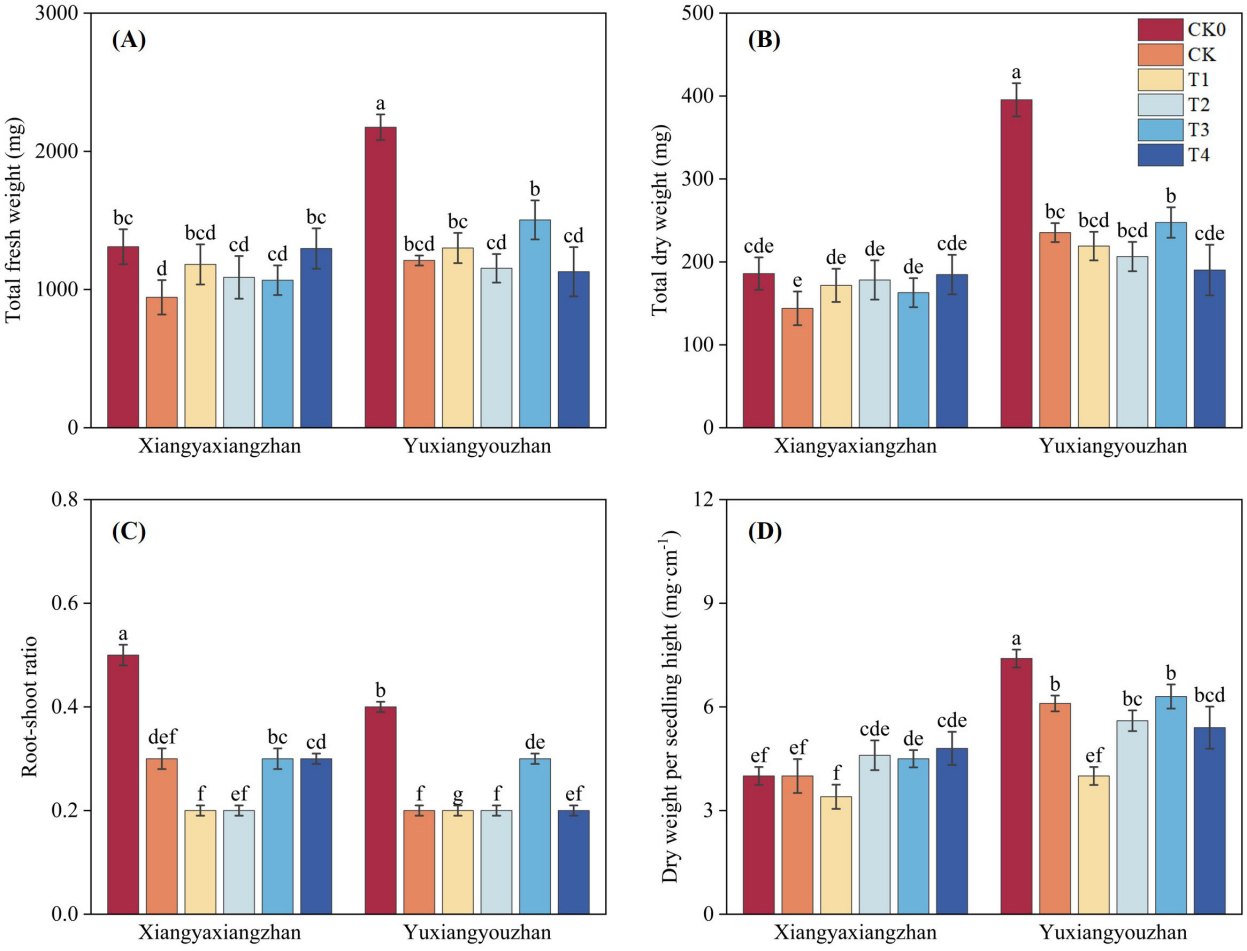
Effects of plant growth regulators and micro-fertilizers on the total biomass and growth parameters under simulated mechanical transplanting injury. Total fresh weight **(A)**, total dry weight **(B)**, root-shoot ratio **(C)**, dry weight per seedling height **(D)**. CK0: Non -injured control, without PGRs or MFs; CK: simulated root-injured control, without PGRs or MFs; T1: 100 mg·L^-1^ gibberellin; T2: 2 mg·L^-1^ indolebutyric acid solution; T3: Mg fertilizers; T4: Zn fertilizers. Lowercase letters indicate significant differences among treatments (LSD test).

The variety, treatment and interaction between variety and treatment significantly affected total fresh weight, total dry weight, root-shoot ratio, and dry weight per seedling height (p < 0.05) (**Table 1**). Compared with CK0, the CK treatment led to reductions in total fresh weight, total dry weight, and root-shoot ratio. In Xiangyaxiangzhan, all four treatments increased total fresh weight relative to CK, with T4 producing a significant increase of 37.41%, whereas in Yuxiangyouzhan, only T1 and T3 enhanced total fresh weight (**Figure 1A**). Similarly, for total dry weight, all four treatments resulted in higher values in Xiangyaxiangzhan compared to CK, while in Yuxiangyouzhan only T3 showed an increase (**Figure 1B**). Regarding the root-shoot ratio, T3 effectively raised 21.43% in Yuxiangyouzhan (**Figure 1C**). For dry weight per seedling height, T2, T3, and T4 led to increases in Xiangyaxiangzhan compare with CK, while in Yuxiangyouzhan only T3 caused an increase (**Figure 1D**).

**Table 1:** AVOVA of the investigated parameters.

Parameter	Variety (V)	Treatment (T)	V×T
Total fresh weight	**	**	**
Total dry weight	**	**	**
Root-shoot ratio	**	**	**
Dry weight per seedling height	**	**	**
Leaf fresh weight	**	**	**
Stem fresh weight	**	**	ns
Root fresh weight	ns	**	**
Shoot fresh weight	**	ns	*
Leaf fresh weight ratio	**	**	**
Stem fresh weight ratio	**	**	**
Root fresh weight ratio	**	**	ns
Leaf dry weight	**	**	**
Stem dry weight	**	**	**
Root dry weight	**	**	**
Shoot dry weight	**	**	**
Leaf dry weight ratio	ns	*	ns
Stem dry weight ratio	**	**	ns
Root dry weight ratio	**	**	*
Shoot dry weight ratio	**	**	*
Plant height	**	**	**
Root length	ns	**	**
Stem length	ns	**	ns
Leaf length	ns	ns	ns
Leaf width	ns	ns	*
Number of green leaves	ns	**	ns
Specific leaf weight	ns	ns	ns
SOD activity in root	ns	**	**
SOD activity in stem	ns	**	*
SOD activity in leaf	ns	**	**
MDA content in root	**	**	**
MDA content in stem	ns	**	**
MDA content in leaf	ns	**	**
CAT activity in root	**	**	**
CAT activity in stem	ns	**	**
CAT activity in leaf	ns	**	**
POD activity in root	**	**	**
POD activity in stem	*	**	**
POD activity in leaf	ns	*	ns

CAT, catalase; SOD, superoxide dismutase; POD, peroxidase; MDA, malondialdehyde. *, significant at p < 0.05; **, significant at p < 0.01; ns, nonsignificant at the p > 0.05 level.

### 3.2 Fresh weight and plant tissue distribution

**Figure 2:**
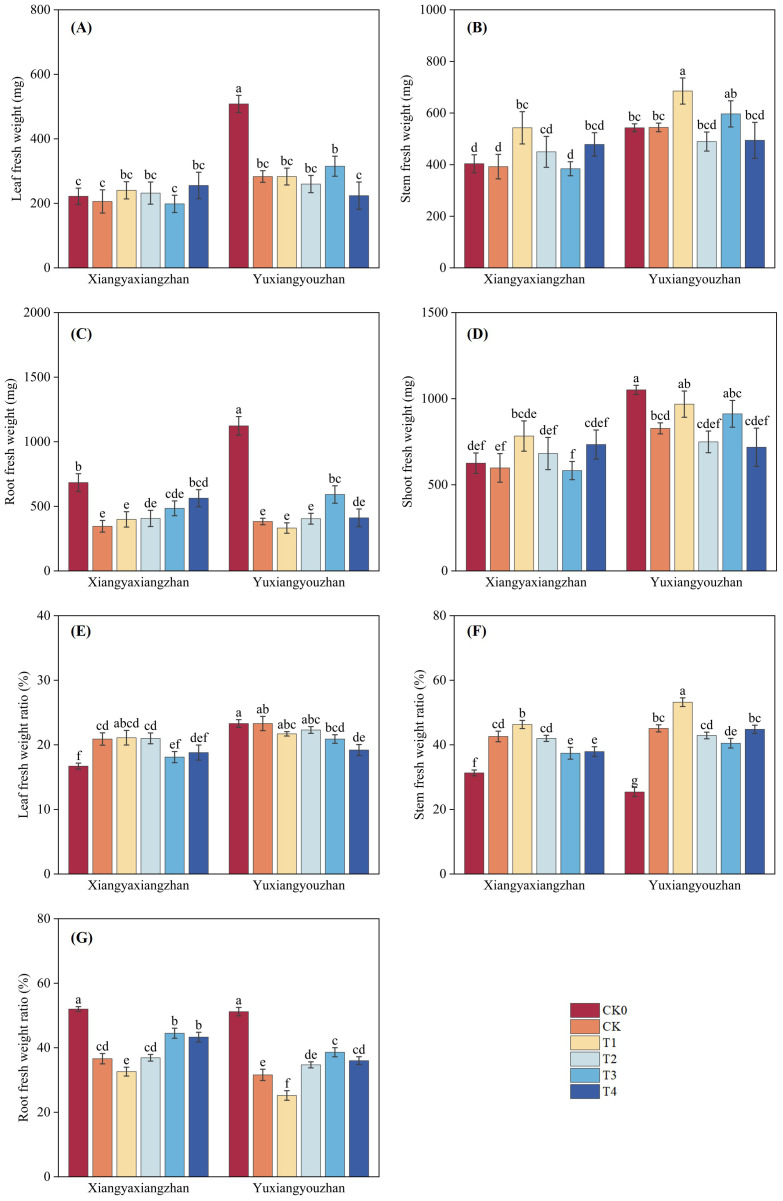
Effects of plant growth regulators and micro-fertilizers on fresh weight and plant tissue distribution under simulated mechanical transplanting injury. Leaf fresh weight **(A)**, Stem fresh weight **(B)**, Root fresh weight **(C)**, Leaf fresh weight ratio **(D)**, Shoot fresh weight **(E)**, Stem fresh weight ratio **(F)**, Root fresh weight ratio **(G)**. CK0: Non -injured control, without PGRs or MFs; CK: simulated root-injured control, without PGRs or MFs; T1: 100 mg·L^-1^ gibberellin; T2: 2 mg·L^-1^ indolebutyric acid solution; T3: Mg fertilizers; T4: Zn fertilizers. Lowercase letters indicate significant differences among treatments (LSD test).

The variety, treatment and interaction between variety and treatment significantly affected leaf fresh weight (p < 0.05) (**Table 1**). As shown in **Figure 2**, compared with CK0, the CK treatment resulted in decreased leaf, stem, and root fresh weight. For Xiangyaxiangzhan, treatments T1, T2, and T4 increased leaf fresh weight relative to CK, whereas in Yuxiangyouzhan, only T3 led to an increase (**Figure 2A**). Regarding stem fresh weight, T1 showing pronounced rise at 38.36%; in Yuxiangyouzhan, T1 being significantly increased the stem fresh weight (**Figure 2B**). For root fresh weight, all four treatments increased it in Xiangyaxiangzhan, while in Yuxiangyouzhan, increases were observed under T2, T3, and T4 (**Figure 2C**). Finally, shoot fresh weight was elevated by T1, T2, and T4 in Xiangyaxiangzhan, and by T1 and T3 in Yuxiangyouzhan (**Figure 2D**).

The variety, treatment and interaction between variety and treatment significantly affected leaf and stem fresh weight (p < 0.05) (**Table 1**). As shown in **Figure 2**, compared with CK0, the CK treatment resulted in increased leaf and stem fresh weight ratios. In Xiangyaxiangzhan, treatments T1 and T2 elevated the leaf fresh weight ratio relative to CK (**Figure 2E**). Regarding the stem fresh weight ratio, T3 and T4 showing significant reductions in in Xiangyaxiangzhan; similarly, in Yuxiangyouzhan, T2 and T3 reduced the stem fresh weight ratio (**Figure 2F**). For root fresh weight ratio, treatments T3 and T4 in Xiangyaxiangzhan produced significant increases of 21.61% and 18.32%, respectively. In Yuxiangyouzhan, T3 significantly increased the root fresh weight ratio by 22.14% (**Figure 2G**).

### 3.3 Dry weight and plant tissue distribution

**Figure 3:**
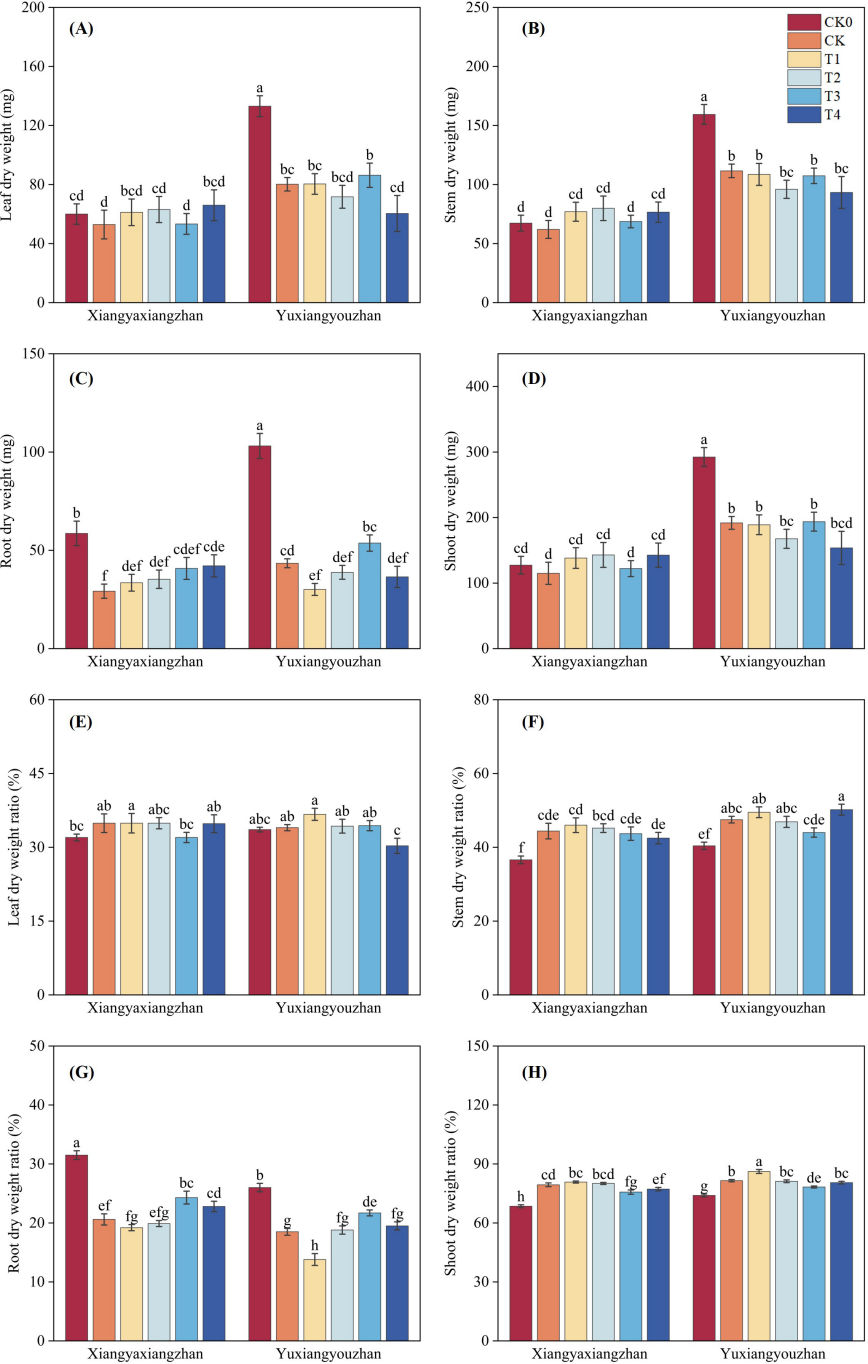
Effects of plant growth regulators and micro-fertilizers on dry weight and plant tissue distribution under simulated mechanical transplanting injury. Leaf dry weight **(A)**, Stem dry weight **(B)**, Root dry weight **(C)**, Shoot dry weight **(D)**, Leaf dry weight ratio **(E)**, Stem dry weight ratio **(F)**, Root dry weight ratio **(G)**, Shoot dry weight ratio **(H)**. CK0: Non -injured control, without PGRs or MFs; CK: simulated root-injured control, without PGRs or MFs; T1: 100 mg·L^-1^ gibberellin; T2: 2 mg·L^-1^ indolebutyric acid solution; T3: Mg fertilizers; T4: Zn fertilizers. Lowercase letters indicate significant differences among treatments (LSD test).

The variety, treatment and interaction between variety and treatment significantly influenced leaf dry weight, stem dry weight, root dry weight, and shoot dry weight (p < 0.05) (**Table 1**). As shown in **Figure 3**, compared with CK0, the CK treatment decreased leaf, stem, root, and shoot dry weight. In Xiangyaxiangzhan, treatments T1, T2, and T4 increased leaf dry weight relative to CK, while in Yuxiangyouzhan, only T3 resulted in an increase (**Figure 3A**). For stem dry weight, all four treatments increased this parameter in Xiangyaxiangzhan compared with CK (**Figure 3B**). Regarding root dry weight, with T4 showing a greatest increase of 44.41% in Xiangyaxiangzhan; in Yuxiangyouzhan, only T3 enhanced root dry weight (**Figure 3C**). Finally, shoot dry weight was increased by all four treatments in Xiangyaxiangzhan (**Figure 3D**).

The variety, treatment and interaction between variety and treatment significantly influenced root and shoot dry weight ratio (p < 0.05) (**Table 1**). As shown in **Figure 3**, compared with CK0, the CK treatment resulted in an increase in leaf dry weight ratio, stem dry weight ratio, and shoot dry weight ratio, while the root dry weight ratio decreased. For Xiangyaxiangzhan, T3 decreased the leaf dry weight ratio compared with CK, whereas for Yuxiangyouzhan, T4 decreased it (**Figure 3E**). In Xiangyaxiangzhan, T3 and T4 decreased the stem dry weight ratio relative to CK; in Yuxiangyouzhan, T2 and T3 decreased the stem dry weight ratio (**Figure 3F**). Regarding the root dry weight ratio, T1 and T2 significantly increased it in Xiangyaxiangzhan compared with CK. In Yuxiangyouzhan, T2, T3, and T4 all increased the root dry weight ratio, with T3 showing the most significant increase of 16.87% (**Figure 3G**). Finally, in Xiangyaxiangzhan, T3 and T4 decreased the shoot dry weight ratio compared with CK (**Figure 3H**).

### 3.4 Plant height, and tissue length and leaf parameters

**Figure 4:**
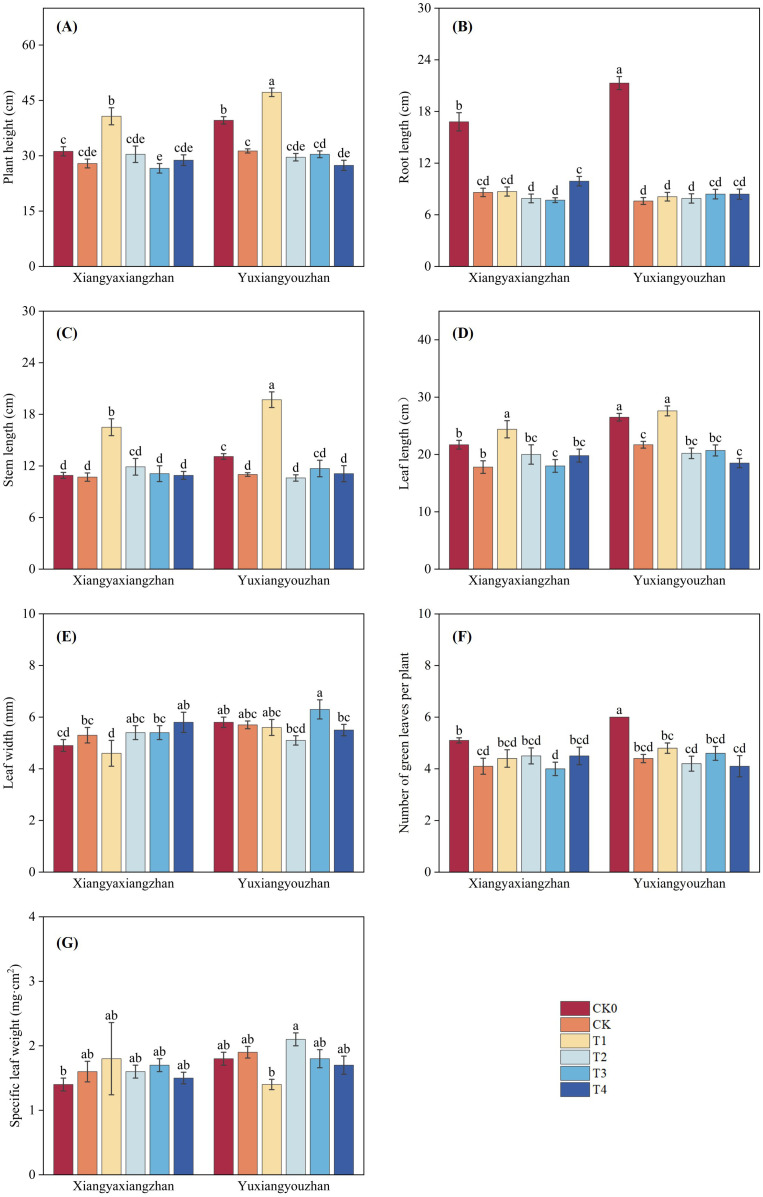
Effects of plant growth regulators and micro-fertilizers on plant height, tissue length and leaf parameters under simulated mechanical transplanting injury. Plant height **(A)**, Root length **(B)**, Stem length **(C)**, Leaf length **(D)**, Leaf width **(E)**, Number of green leaves **(F)**, Specific leaf weight **(G)**. CK0: Non -injured control, without PGRs or MFs; CK: simulated root-injured control, without PGRs or MFs; T1: 100 mg·L^-1^ gibberellin; T2: 2 mg·L^-1^ indolebutyric acid solution; T3: Mg fertilizers; T4: Zn fertilizers. Lowercase letters indicate significant differences among treatments (LSD test).

The variety, treatment and interaction between variety and treatment significantly affected plant height (p < 0.05) (**Table 1**). As shown in **Figure 4**, compared with CK0, the CK treatment resulted in decreases in plant height, root length, stem length, leaf length, and the number of green leaves. In Xiangyaxiangzhan, T1 significantly increased seedling plant height by 45.94%; in Yuxiangyouzhan, T1 significantly increased seedling plant height by 50.91% (**Figure 4A**). For root length, T4 increased this parameter in Xiangyaxiangzhan compared with CK, while all four treatments increased root length in Yuxiangyouzhan (**Figure 4B**). Regarding stem length, T1 showing a significant increase of 53.49%; in Yuxiangyouzhan, T1 significantly increased seedling stem length by 79.01% (**Figure 4C**). For leaf length, T1 showing a great increase of 37.09%; in Yuxiangyouzhan, only T1 increased leaf length (**Figure 4D**). In terms of leaf width, treatments T2, T3, and T4 increased it in Xiangyaxiangzhan, whereas in Yuxiangyouzhan only T3 resulted in an increase (**Figure 4E**). Finally, the number of green leaves was increased by T1, T2, and T4 in Xiangyaxiangzhan, and by T1 and T3 in Yuxiangyouzhan (**Figure 4F**).

### 3.5 Antioxidant attributes

**Figure 5:**
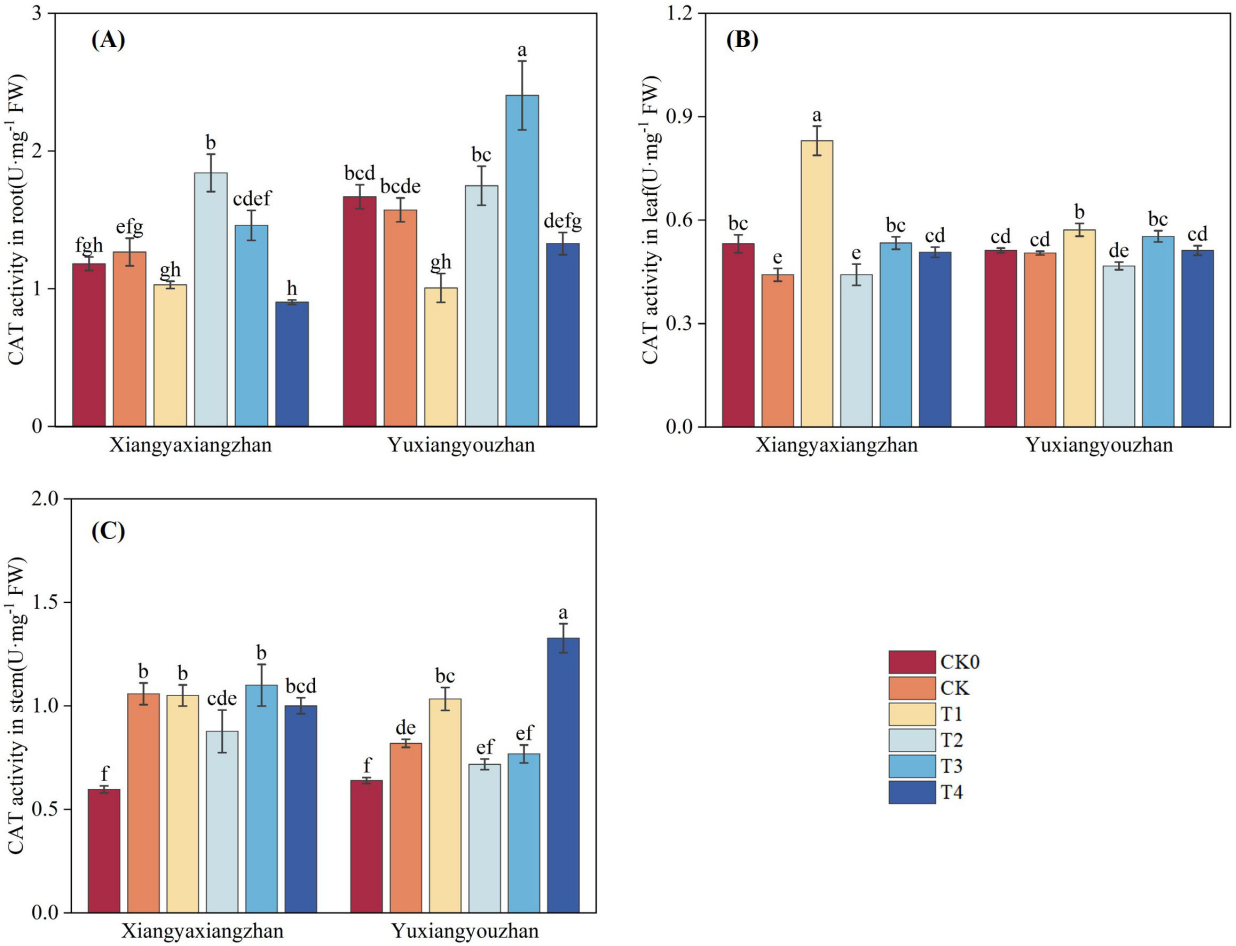
Effects of plant growth regulators and micro-fertilizers on catalase (CAT) activity in rice seedlings under simulated transplanting injury. CAT activity in root **(A)**, CAT activity in leaf **(B)**, CAT activity in stem **(C)**. CK0: Non -injured control, without PGRs or MFs; CK: simulated root-injured control, without PGRs or MFs; T1: 100 mg·L^-1^ gibberellin; T2: 2 mg·L^-1^ indolebutyric acid solution; T3: Mg fertilizers; T4: Zn fertilizers. Different lowercase letters indicate significant differences among treatments (LSD test).

The variety, treatment and interaction between variety and treatment significantly affected CAT activity in root (p < 0.05) (**Table 1**). As shown in **Figure 5**, compared with CK0, the CK treatment increased CAT activity in both root and stem of Xiangyaxiangzhan, whereas in Yuxiangyouzhan, CAT activity increased only in stem. In Xiangyaxiangzhan, compared with CK, T1 significantly increased leaf CAT activity by 87.97%, while T2 enhanced root CAT activity by 45.35%. T3 noticeable elevated CAT activity in leaf by 20.83%. In Yuxiangyouzhan, relative to CK, T1 significantly raised CAT activity in stem and leaf by 26.15% and 13.37%, respectively. T3 significantly improved CAT activity in root by 52.86%. T4 increased CAT activity in stem by 62.00%.

**Figure 6:**
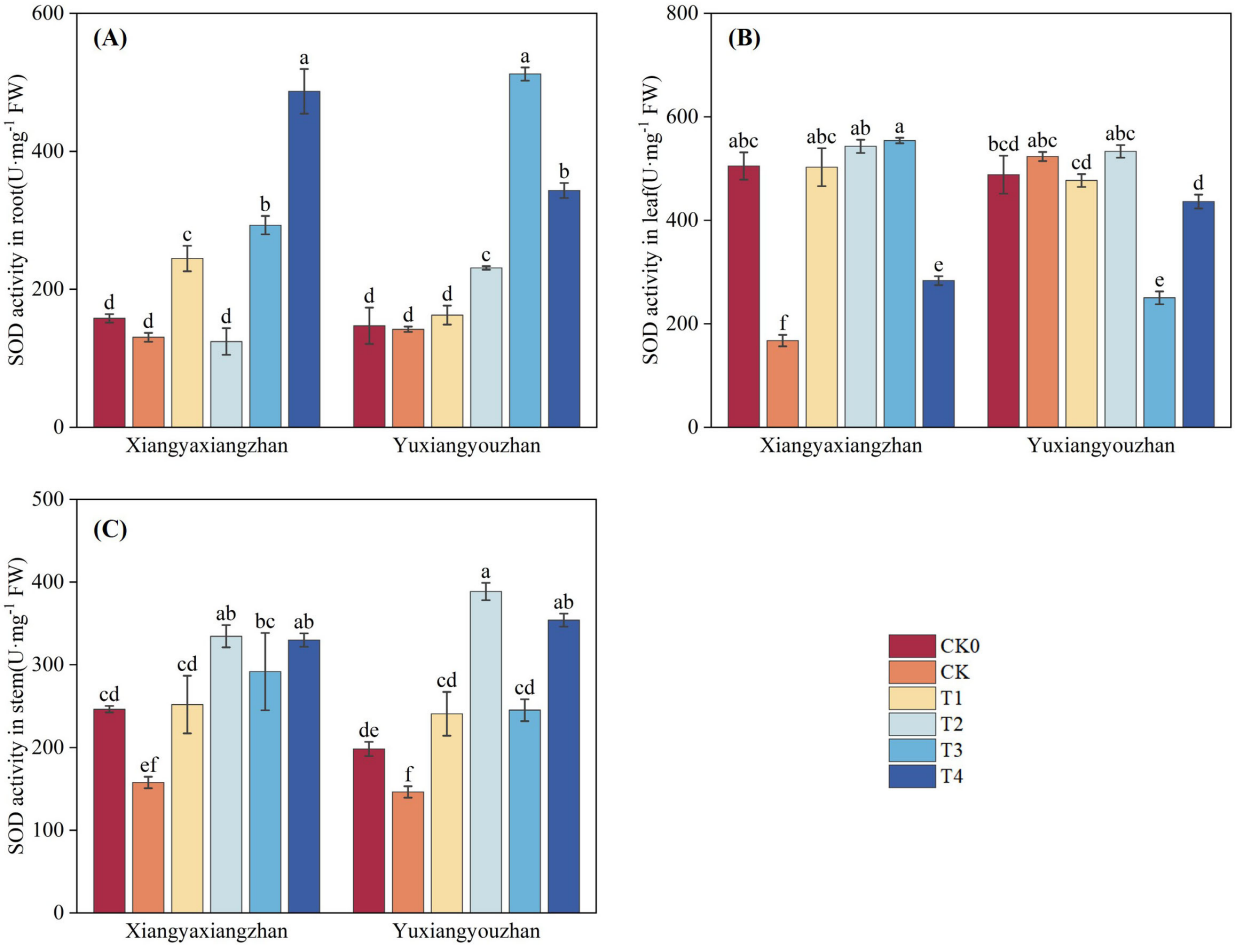
Effects of plant growth regulators and micro-fertilizers on superoxide dismutase (SOD) activity in rice seedlings under simulated transplanting injury. SOD activity in root **(A)**, SOD activity in leaf **(B)**, SOD activity in stem **(C)**. CK0: Non -injured control, without PGRs or MFs; CK: simulated root-injured control, without PGRs or MFs; T1: 100 mg·L^-1^ gibberellin; T2: 2 mg·L^-1^ indolebutyric acid solution; T3: Mg fertilizers; T4: Zn fertilizers. Different lowercase letters indicate significant differences among treatments (LSD test).

The variety, treatment and interaction between variety and treatment significantly affected SOD activity in root, stem and leaf (p < 0.05) (**Table 1**). As shown in **Figure 6**, compared with CK0, only Yuxiangyouzhan exhibited increased leaf SOD activity under the CK treatment. In Xiangyaxiangzhan, T1 significantly increased SOD activity in root, stem, and leaf by 87.52%, 59.77%, and 199.74%, respectively. T2 significantly enhanced SOD activity in stem and leaf by 112.15% and 223.77%. T3 significantly elevated SOD activity in all tissues, with increases of 124.57% in root, 85.08% in stem, and 230.49% in leaf. T4 also significantly increased SOD activity in root, stem, and leaf by 273.27%, 109.23%, and 69.11%, respectively. In Yuxiangyouzhan, T1 significantly increase stem SOD activity by 64.50%. T2 significantly increase SOD activity by 62.51% in root and 165.63% in stem, respectively. T3 significantly improved SOD activity in root and stem by 260.22% and 67.52%, respectively. T4 significantly increased SOD activity in root and stem by 141.49% and 141.88%.

**Figure 7:**
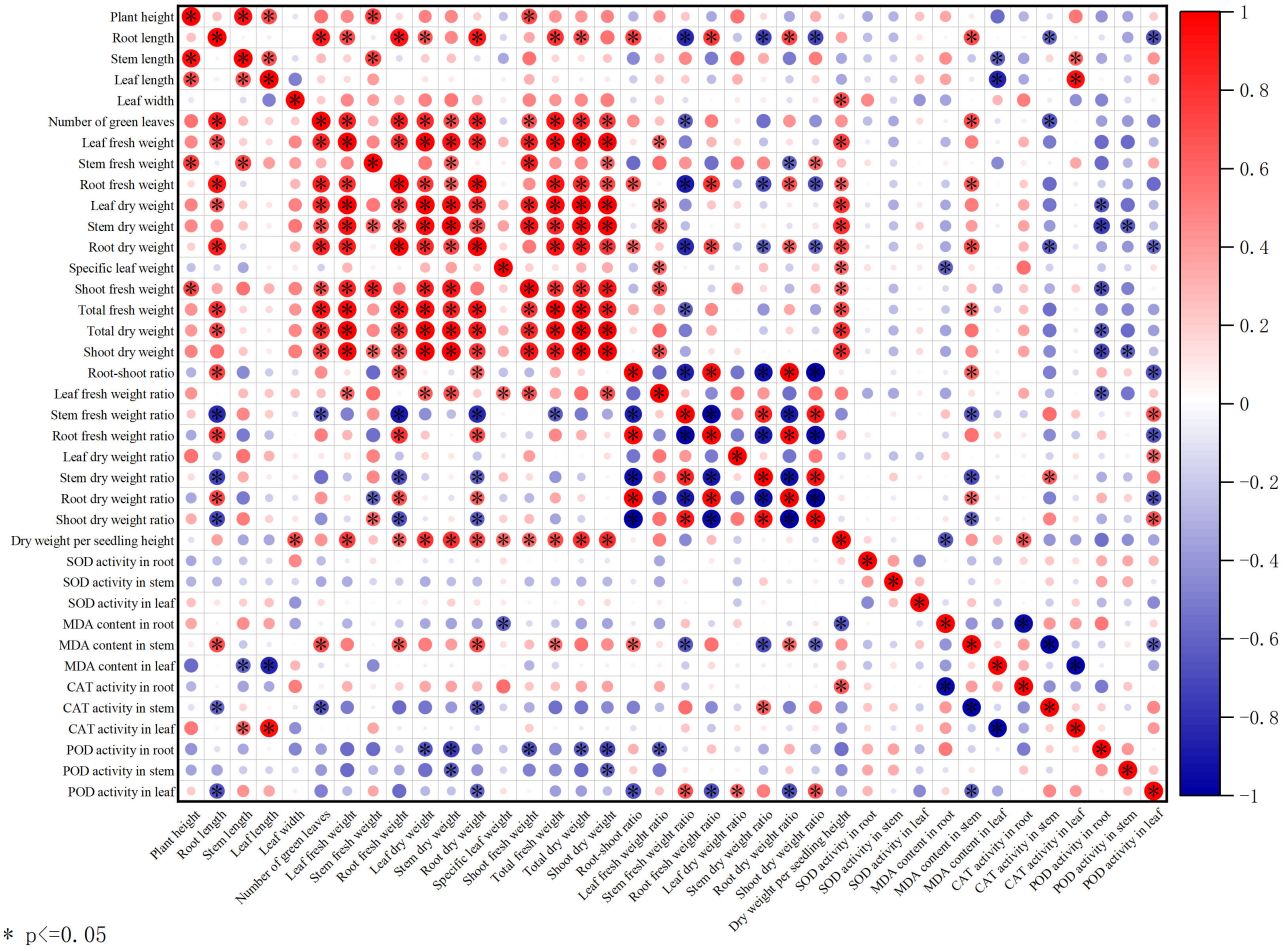
Correlation analysis between the investigated parameters.

The variety, treatment and interaction between variety and treatment significantly affected POD activity in root, stem (p < 0.05) (**Table 1**). As shown in **Figure 7**, compared with CK0, POD activity increased in the leaf of Xiangyaxiangzhan and in root, stem, and leaf of Yuxiangyouzhan under the CK treatment. In Xiangyaxiangzhan, T4 significantly increased POD activity in root by 33.47%. In Yuxiangyouzhan, T1 significantly increased POD activity in stem by 26.73%. T2 significantly increasing activity in root and stem by 54.33% and 51.59%, respectively. T3 significantly increased POD activity in stem by 72.73%. T4 significantly increased POD activity in root by 38.77%.

**Figure 8:**
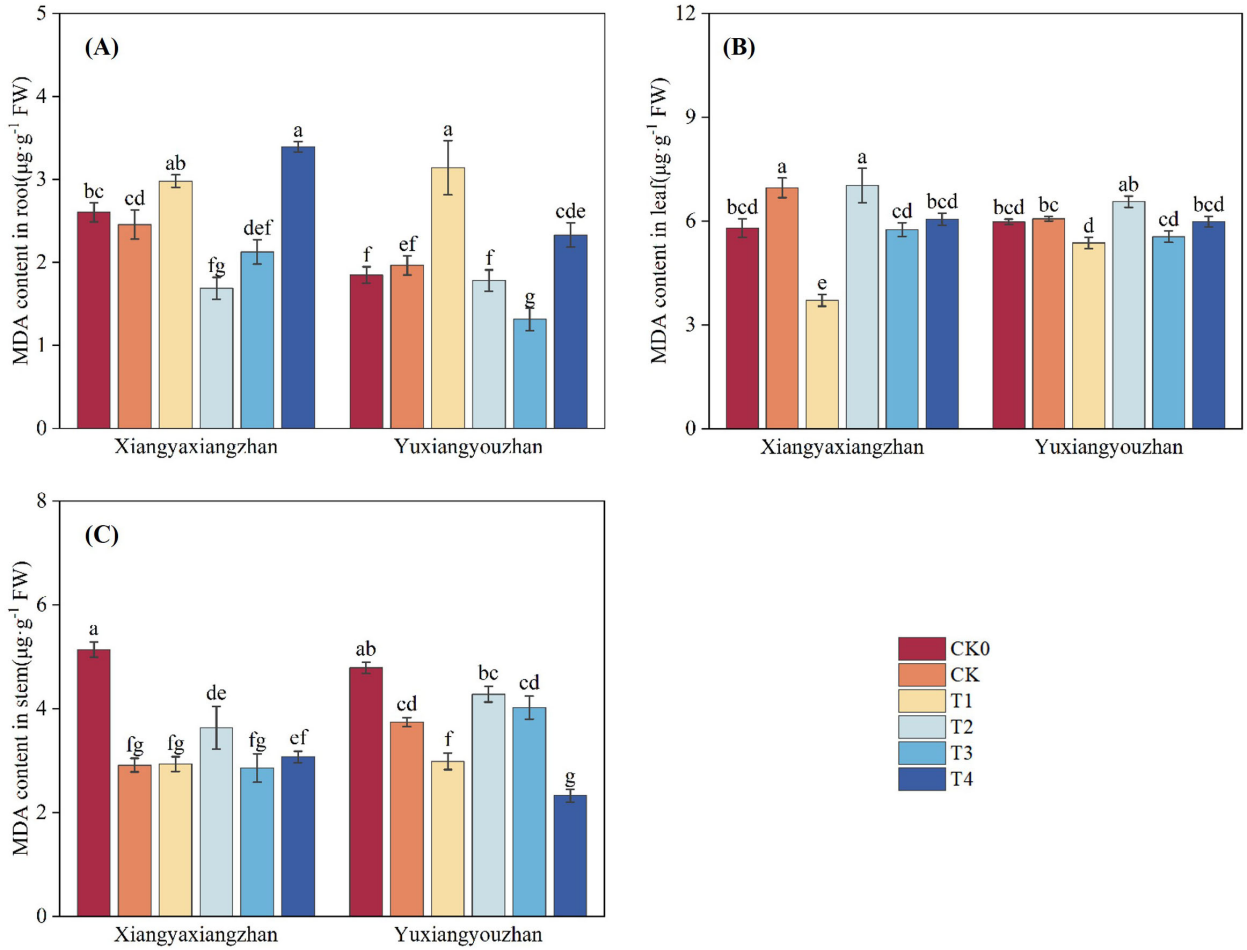
Effects of plant growth regulators and micro-fertilizers on malonaldehyde (MDA) content in rice seedlings under simulated transplanting injury. MDA content in root **(A)**, MDA content in leaf **(B)**, MDA content in stem **(C)**. CK0: Non -injured control, without PGRs or MFs; CK: simulated root-injured control, without PGRs or MFs; T1: 100 mg·L^-1^ gibberellin; T2: 2 mg·L^-1^ indolebutyric acid; T3: Mg fertilizers; T4: Zn fertilizers. Lowercase letters indicate significant differences among treatments (LSD test).

The variety significantly affected MDA content in root (p < 0.05). Treatment had a significant effect on MDA content in root, stem, and leaf (p < 0.05). The interaction between variety and treatment significantly affected MDA content in stem and leaf (p < 0.05) (**Table 1**). As shown in **Figure 8**, compared with CK0, MDA content decreased in the root and stem of Xiangyaxiangzhan and in the stem of Yuxiangyouzhan under the CK treatment. In Xiangyaxiangzhan, T1 significantly reduced MDA content in leaf by 46.70%. T2 significantly decreased MDA content in root by 31.19%. T4 significantly lowered MDA content in leaf by 13.11%. In Yuxiangyouzhan, T1 significantly reduced MDA content in stem and leaf by 20.18% and 11.57%, respectively. T4 significantly decreased MDA content in stem by37.58%.

### 3.6 Correlation analysis

**Figure 9:**
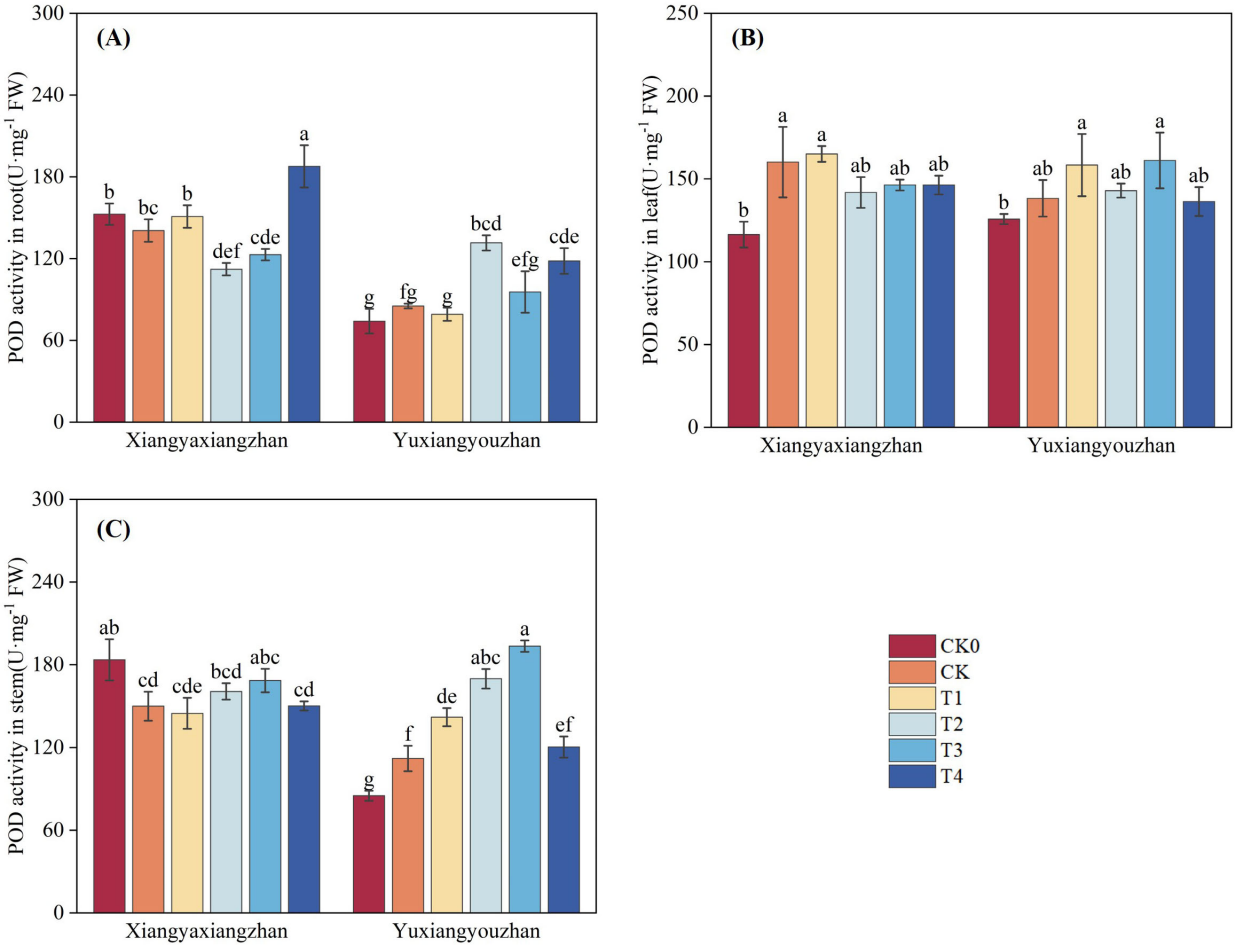
Effects of plant growth regulators and micro-fertilizers on peroxidase (POD) activity in rice seedlings under simulated transplanting injury. POD activity in root **(A)**, POD activity in leaf **(B)**, POD activity in stem **(C)**. CK0: Non -injured control, without PGRs or MFs; CK: simulated root-injured control, without PGRs or MFs; T1: 100 mg·L^-1^ gibberellin; T2: 2 mg·L^-1^ indolebutyric acid solution; T3: Mg fertilizers; T4: Zn fertilizers. Lowercase letters indicate significant differences among treatments (LSD test).

**Figure 9** show that total dry weight was positively correlated with plant height, root length, number of green leaves, leaf fresh weight, and stem fresh weight, while it was negatively correlated with root−shoot ratio, leaf dry weight ratio, and leaf MDA content. Root POD activity was positively correlated with stem dry weight, root dry weight, and root CAT activity, and negatively correlated with MDA content in both root and stem. These findings further highlight the link between improved seedling growth and enhanced root POD activity.

## 4 Discussion

Root cell damage inhibits the growth of rice seedlings (Zhang et al., 2017). Indicators such as root length, total fresh weight, and total dry weight are important metrics for assessing root development (Figueroa-Bustos et al., 2018; Halder et al., 2024), while the number of green leaves, leaf fresh weight, and leaf dry weight are key parameters for evaluating overall plant growth performance (Zhu et–al., 2020), which is consistent with the findings of this study. In the present study, compared to the control group (CK), the CK0, where root pruning was used to simulate transplanting injury, exhibited significant reductions not only in root length, total dry weight, total fresh weight, and root fresh weight, but also in shoot growth indicators such as the number of green leaves, leaf fresh weight, and leaf dry weight.

Multiple researchers have reported positive effects of foliar application of gibberellin on various field crops. Gibberellin-treated maize showed increased plant height and improved growth status (Shahzad et al., 2021). Seed priming with GA_3_ could increase the seedling height at the seedling stage and significantly thicken the stem diameter of soybean at harvest stage (Han et al., 2021). In present study, foliar application of gibberellin promoted the growth of rice seedlings, as evidenced by significant improvements in plant height, leaf length, and stem length (**Figure 4**). This may be attributed to gibberellin being a key hormone in cell division, promoting cell elongation growth. Furthermore, gibberellin significantly improved the performance of the shoot of rice seedlings in this study, such as shoot fresh weight and stem fresh weight. This could be due to the foliar application of gibberellin increasing the synthesis of various metabolites such as sugars, free amino acids, proline, and phenolics (Mohamed and Faisal, 2021) and being associated with increased stem water content and water retention capacity (Islam et al., 2023). This aligns with the findings of Islam et al. (2023), who reported that gibberellin significantly improves water relations, morphology, and yield attributes in mung bean. The potential role of promoted endogenous accumulation following exogenous gibberellin supply may also be involved (Iftikhar et al., 2019).

Previous studies have shown that mechanical transplanting injury generates more reactive oxygen species (ROS), causing damage to seedlings (Li et al., 2016). Therefore, investigating the restoration of the antioxidant enzyme system under transplanting injury is particularly important. SOD, CAT, and POD are core enzymes in the plant antioxidant enzyme system, and increased their activities can enhance the plant’s ability to scavenge ROS; MDA is a key product of membrane lipid peroxidation, and a decrease in its content can reflect reduced damage to the plant membrane system. In present study, after foliar application of gibberellin, the CAT activity in the stem and leaf and the SOD activity in the stem of rice seedlings increased significantly, while the activities of related enzymes in the root decreased. This indicates that gibberellin can mitigate the adverse effects of root damage on the plant’s antioxidant defense system by regulating the activities of antioxidant enzymes, thereby supporting seedling injury repair and compensatory growth (Bhat et al., 2023).

Previous studies have reported positive effects of Mg fertilizer foliar application on crops. For instance, it significantly increased N, K, and Ca accumulation, panicle weight, and grain fresh weight in maize (Ye et al., 2025); it increased trace element and complex compound content in rice, positively affecting yield and quality (He et al., 2024). In present study, foliar application of Mg fertilizer improved the overall growth performance of the Xiangyaxiangzhan during the recovery stage from transplanting injury. This may be due to the foliar application of Mg fertilizer improving the overall nutrient uptake and enhancing carbohydrate accumulation in rice seedlings (Ye et al., 2025). This primarily contributed to improvements in total fresh weight and total dry weight (**Figure 1**). The lack of a clear effect of Mg fertilizer foliar application on improving the growth performance of the Yuxiangyouzhan might require further research to elucidate the reasons.

Furthermore, foliar application of Mg fertilizer significantly increased CAT activity in leaf, SOD activity in root and leaf, and POD activity in root under simulated mechanical transplanting injury conditions, and significantly reduced MDA content. This indicates that foliar application of Mg fertilizer can enhance antioxidant enzyme activities (Santos et al., 2025) and reduce MDA content, thereby mitigating the adverse effects of mechanical transplanting injury on rice seedlings.

Zinc is an essential micronutrient for the normal growth and development of rice, serving as a cofactor for various enzymatic reactions including auxin and chlorophyll synthesis (Dang et al., 2025). Zinc deficiency in rice leads to stunted plants, inhibited leaf growth accompanied by chlorosis, delayed heading and reduced panicle, which severely affects yield (Hafeez et al., 2013; Tuiwong et al., 2021). Research has shown that foliar application of zinc sulfate at 20 and 40 days after sowing can improve plant height, leaf area index, leaf weight ratio, leaf area ratio, specific leaf weight, crop growth rate, relative growth rate, and net assimilation rate in sweet corn, positively influencing crop growth and development (Kaur et al., 2024). The study by Ana R. A. de Moraes et al. indicated that Zn fertilization promoted increased leaf area, height, shoot dry weight, and total dry weight in dwarf green coconut seedlings. Meanwhile, Zn fertilization improved photosynthesis, transpiration, and enhanced seedling growth and photosynthetic capacity (de Moraes et al., 2023). Under drought stress, foliar application of Si and Zn fertilizer can increase key growth parameters such as plant height, leaf area, chlorophyll content, and yield in maize, alleviating the effects of drought stress (Lamlom et al., 2024). Application of Zn fertilizer reduced hydrogen peroxide content and increased CAT activity in citrus (Boaretto et al., 2024); under salt stress, foliar application of Zn fertilizer significantly enhanced the activities of antioxidant enzymes such as SOD, POD, and CAT in barley (Noreen et al., 2021). Additionally, research by Wu et al. showed that foliar application of Zn and Se fertilizer reduced MDA content in wheat root (Wu et al., 2020). In the present study, foliar application of Zn fertilizer increased seedling shoots fresh weight, root fresh weight ratio, CAT activity in stem and leaf, SOD activity in root and stem, and POD activity in root, while decreasing MDA content in stem and leaf. The increases in SOD activity in stem and POD activity in root were particularly significant. We concur that the application of Zn fertilizer can enhance antioxidant enzyme activities and reduce ROS-related metabolites. We propose that foliar application of Zn fertilizer increases antioxidant enzyme activities because zinc is an essential component of SOD (Doloksaribu et al., 2024). After foliar absorption, the zinc ion content in the plant increases, allowing more zinc to bind to the active site of SOD, promoting its activation (Kamran et al., 2023). SOD is positioned at the upstream of the ROS scavenging pathway, and plants prioritize enhancing SOD activity to rapidly control the initial ROS burst (Jia et al., 2022). Therefore, under sufficient zinc supply, the response of SOD activity is most sensitive and significant, especially in metabolically active stem like the stem. The enhanced activity of SOD and other antioxidant enzymes leads to efficient scavenging of ROS, reducing the attack on cell membranes and consequently decreasing the degree of lipid peroxidation, thereby lowering MDA content (**Figures 5**, **6**, **9**, **8**).

Auxin is a trace substance that significantly influences plant growth, development, and physiological biochemistry. Auxin plays crucial roles in promoting cell division, elongation, the differentiation and formation of new organs, and overall plant growth and development (Paque and Weijers, 2016). Application of indolebutyric acid during the rice seedling stage can promote root differentiation, dwarf seedlings, strengthen seedlings, increase effective tillering, enhance stress resistance, and improve yield (Wahyuni et al., 2016). indolebutyric acid-derived auxin exerts strong effects on various aspects of root development, including regulating root apical meristem size, root hair elongation, lateral root development, and adventitious root formation, and is commonly used to promote adventitious root growth in cuttings of horticultural plants (El-Banna et al., 2023; Frick and Strader, 2018). Studies have shown that seed priming with indolebutyric acid significantly increased shoot and root fresh and dry weight, shoot and root length, and leaf number; indolebutyric acid can be used to improve morphological, biochemical, and yield parameters in wheat (Bashir et al., 2021). Foliar application of exogenous indolebutyric acid increased root, shoot, and whole plant biomass in Stellaria media, and improved photosynthetic pigment content, antioxidant enzyme activities, soluble protein content, and soluble sugar content (Lin L, et al., 2024). Under 20 mg·L^-^¹ salt stress, seed soaking with IBA-K significantly increased plant height in two rapeseed varieties, enhanced the activities of antioxidant enzymes such as SOD, POD, and CAT, while significantly reducing the levels of electrolyte leakage and MDA (Li et al., 2024). In the present study, foliar application of indolebutyric acid increased seedling root fresh weight, root fresh weight ratio, CAT activity in root, SOD activity in root and stem, and POD activity in stem, while decreasing MDA content in root. The effects on CAT activity in root and POD activity in stem were particularly significant, and the effects were more pronounced in the Xiangyaxiangzhan (**Figures 5**, **6**, **9**, **8**).

**Figure 10:**
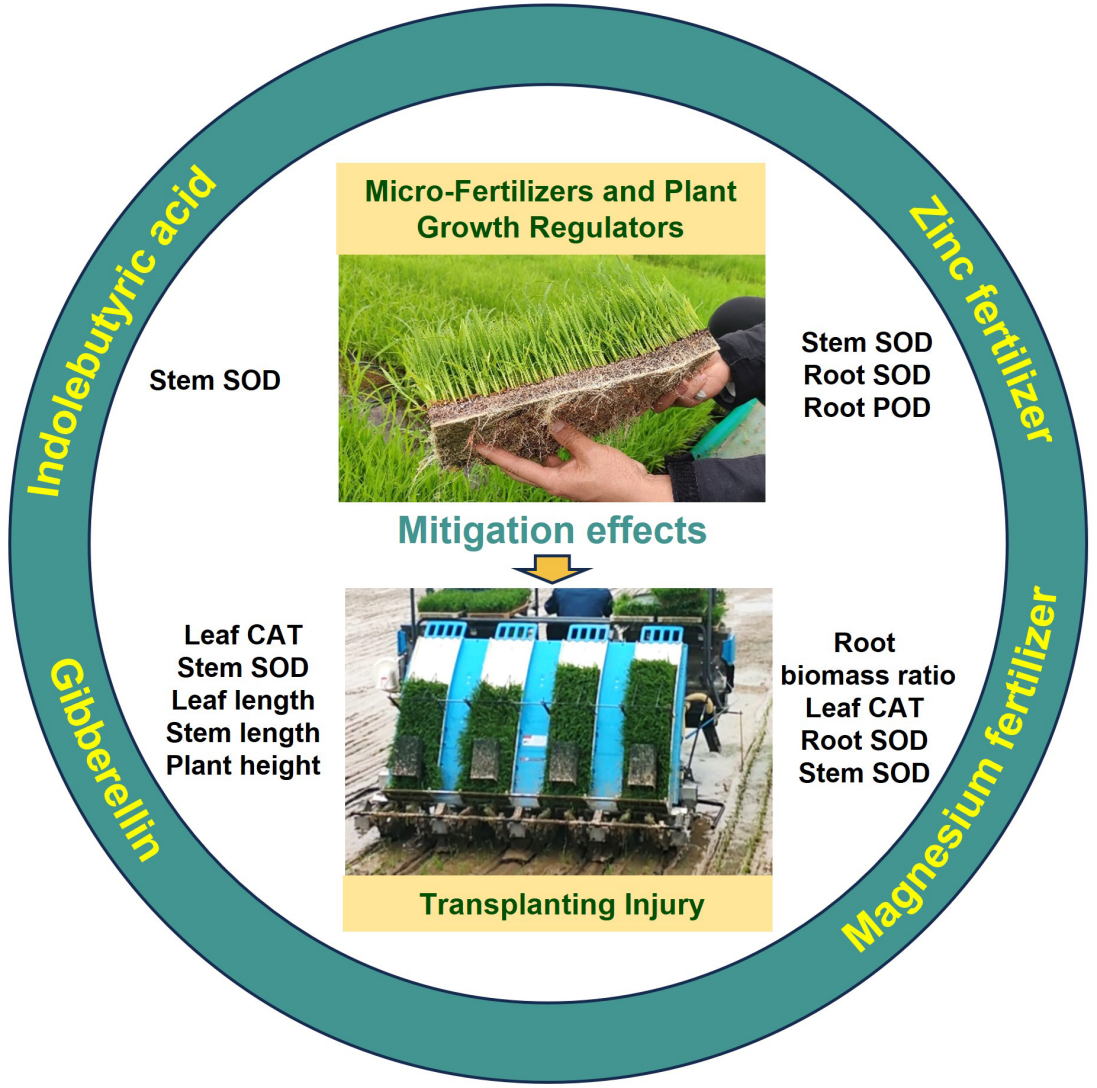
Unraveling the mechanisms by which plant growth regulators and micro-fertilizers enhance recovery of fragrant rice seedlings from transplanting injury.

Overall, the application of micro-fertilizers and plant growth regulators alleviates transplanting injury in plants by regulating key morphological and physiological indicators. Specifically: indolebutyric acid enhances stem SOD activity; gibberellin improves multiple traits, including stem fresh weight, leaf length, plant height, leaf CAT activity, and leaf MDA content; zinc fertilizer modulates SOD activity in roots and stems, as well as root POD activity; and magnesium fertilizer increases the root biomass ratio, root−shoot ratio, and SOD activity in roots and stems. The significant improvement in these parameters confirms the efficacy of these treatments in mitigating transplanting injury (**Figure 10**).

## 5 Conclusion

This study systematically elucidates the distinct physiological mechanisms through which plant growth regulators (PGRs) and micro-fertilizers (MFs) alleviate transplanting injury in fragrant rice. The results indicate that GA primarily accelerates seedling recovery by activating shoot growth and the antioxidant system; IBA specifically enhances stem SOD activity to establish localized defense; whereas Mg and Zn fertilizers synergistically improve root architecture and upregulate SOD/POD activities across multiple tissues, thereby establishing systemic stress resistance. Notably, Mg fertilizer demonstrated the most comprehensive benefits by optimizing root-shoot allocation and robustly enhancing SOD activity. In conclusion, modulating antioxidant pathways can effectively mitigate transplanting injury, and future research should further elucidate the regulatory mechanisms of root-shoot interaction in this process.

## Data Availability

The original contributions presented in the study are included in the article/supplementary material. Further inquiries can be directed to the corresponding author.

## References

[B1] Bashir Z. Hussain K. Iqbal I. Nawaz K. Siddiqui E. H. Javeria M. . (2021). Assessment of growth, yield and economic analysis of wheat (Triticum aestivum L.) by plant growth regulators. Pak J. Bot. 53, 585–595. doi: 10.30848/PJB2021-2(3)

[B2] Bhat J. A. Basit F. AlYemeni M. N. Mansoor S. Kaya C. Ahmad P. (2023). Gibberellic acid mitigates nickel stress in soybean by cell wall fixation and regulating oxidative stress metabolism and glyoxalase system. Plant Physiol. Biochem. 198, 107678. doi: 10.1016/j.plaphy.2023.107678, PMID: 37054613

[B3] Boaretto R. M. Hippler F. W. R. Teixeira L. A. J. Fornari R. C. Quaggio J. A. Mattos D. (2024). Zinc fertilizers for citrus production: assessing nutrient supply via fertigation or foliar application. Plant Soil 496, 179–192. doi: 10.1007/s11104-023-05969-w

[B4] Chen W. Liao G. Sun F. Ma Y. Chen Z. Chen H. . (2023). Foliar spray of La2O3 nanoparticles regulates the growth, antioxidant parameters, and nitrogen metabolism of fragrant rice seedlings in wet and dry nurseries. Environ. Sci. pollut. Res. 30, 80349–80363. doi: 10.1007/s11356-023-27892-4, PMID: 37296245

[B5] Chen S. Liu S. Zheng X. Yin M. Chu G. Xu C. . (2018). Effect of various crop rotations on rice yield and nitrogen use efficiency in paddy–upland systems in southeastern China. Crop J. 6, 576–588. doi: 10.1016/j.cj.2018.07.007

[B6] Chou T. Chao Y. Huang W. Hong C. Kao C. (2011). Effect of magnesium deficiency on antioxidant status and cadmium toxicity in rice seedlings. J. PlantPhysiol. 168, 1021–1030. doi: 10.1016/j.jplph.2010.12.004, PMID: 21216027

[B7] Dang K. Tian H. Bai J. Fu P. Cui J. Ji D. . (2025). Ameliorating effect of zinc on water transport in rice plants under saline-sodic stress. Front. Plant Sci. 16, 1616333. doi: 10.3389/fpls.2025.1616333, PMID: 40894493 PMC12391086

[B8] Danso Ofori A. Zheng T. Titriku J. K. Appiah C. Xiang X. Kandhro A. G. . (2025). The role of genetic resistance in rice disease management. Int. J. Mol. Sci. 26, 956. doi: 10.3390/ijms26030956, PMID: 39940724 PMC11817016

[B9] de Moraes A. R. A. Araújo S. R. da Silva Junior M. L. Lins P. M. P. Gomes M. S. Gomes M. F. . (2023). Fertilization with zinc improves the growth and photosynthetic performance of dwarf green coconut seedlings. Rev. Bras. Eng. Agríc E Ambient 27, 864–872. doi: 10.1590/1807-1929/agriambi.v27n11p864-872

[B10] Doloksaribu B. Siahaan G. Lestrina D. Sari Y. D. Anjany D. (2024). Correlation between antioxidant intake with pro-oxidants (MDA) and endogenous antioxidants (SOD) in football athletes. J. Pendidik Jasm Dan Olahraga 9, 161–168. doi: 10.17509/jpjo.v9i2.63202

[B11] Drummond I. Austin-Tse C. (2013). “ Zebrafish Cilia,” in Methods Enzymol, vol. 525. ( Elsevier), 219–244. 23522472 10.1016/B978-0-12-397944-5.00011-0

[B12] El-Banna M. F. Farag N. B. B. Massoud H. Y. Kasem M. M. (2023). Exogenous IBA stimulated adventitious root formation of Zanthoxylum beecheyanum K. Koch stem cutting: histo-physiological and phytohormonal investigation. Plant Physiol. Biochem. 197, 107639. doi: 10.1016/j.plaphy.2023.107639, PMID: 36989985

[B13] Figueroa-Bustos V. Palta J. A. Chen Y. Siddique K. H. M. (2018). Characterization of root and shoot traits in Wheat cultivars with putative differences in root system size. Agronomy 8, 109. doi: 10.3390/agronomy8070109

[B14] Frick E. M. Strader L. C. (2018). Roles for IBA-derived auxin in plant development. J. Exp. Bot. 69, 169–177. doi: 10.1093/jxb/erx298, PMID: 28992091 PMC5853464

[B15] Hafeez B. Khanif Y. M. Saleem M. (2013). Role of zinc in plant nutrition - a review. Am. J. Exp. Agric. 3, 374–391. doi: 10.9734/AJEA/2013/2746

[B16] Halder T. Stroeher E. Liu H. Chen Y. Yan G. Siddique K. H. M. (2024). Protein biomarkers for root length and root dry mass on chromosomes 4A and 7A in wheat. J. Proteomics 291, 105044. doi: 10.1016/j.jprot.2023.105044, PMID: 37931703

[B17] Han Y. Shi Y. Gao Y. Zheng D. Du J. Zhang Y. . (2021). Effects of gibberellins and uniconazole on morphology, photosynthetic physiology, and yield of soybean. Chin. J. Oil Crop Sci. 43, 651–659. doi: 10.7505/j.issn.1007_9084.2018.06.011

[B18] He Z. Wang Z. Hao J. Wu Y. Liu H. (2024). The use of magnesium fertilizer can improve the nutrient uptake, yield, and quality of rice in liaoning province. Agronomy 14, 639. doi: 10.3390/agronomy14030639

[B19] He W. Zhong Q. He B. Wu B. Mohi Ud Din A. Han J. . (2022). N-acetylcysteine priming alleviates the transplanting injury of machine-transplanted rice by comprehensively promoting antioxidant and photosynthetic systems. Plants 11, 1311. doi: 10.3390/plants11101311, PMID: 35631736 PMC9144612

[B20] Hong W. Chen Y. Huang S. Li Y. Wang Z. Tang X. . (2022). Optimization of nitrogen–silicon (N-Si) fertilization for grain yield and lodging resistance of early-season indica fragrant rice under different planting methods. Eur. J. Agron. 136, 126508. doi: 10.1016/j.eja.2022.126508

[B21] Huang Z. Xie W. Wang M. Liu X. Ashraf U. Qin D. . (2020). Response of rice genotypes with differential nitrate reductase-dependent NO synthesis to melatonin under ZnO nanoparticles’ (NPs) stress. Chemosphere 250, 126337. doi: 10.1016/j.chemosphere.2020.126337, PMID: 32135442

[B22] Iftikhar A. Ali S. Yasmeen T. Arif M. S. Zubair M. Rizwan M. . (2019). Effect of gibberellic acid on growth, photosynthesis and antioxidant defense system of wheat under zinc oxide nanoparticle stress. Environ. pollut. 254, 113109. doi: 10.1016/j.envpol.2019.113109, PMID: 31487671

[B23] Islam M. S. Hasan M. Islam M. Chowdhury M. Pramanik M. H. Iqbal M. A. . (2023). Water relations and yield characteristics of mungbean as influenced by foliar application of gibberellic acid (GA3). Front. Ecol. Evol. 11, 1048768. doi: 10.3389/fevo.2023.1048768

[B24] Jia P. Melnyk A. Li L. Kong X. Dai H. Zhang Z. (2022). Differential adaptation of roots and shoots to salt stress correlates with the antioxidant capacity in mustard (Brassica juncea L.). Pak J. Bot. 54, 2001–2011. doi: 10.30848/PJB2022-6(32)

[B25] Kamran A. Ghazanfar M. Khan J. S. Pervaiz S. Siddiqui M. H. Alamri S. (2023). Zinc Absorption through Leaves and Subsequent Translocation to the Grains of Bread Wheat after Foliar Spray. Agriculture 13, 1775. doi: 10.3390/agriculture13091775

[B26] Kaur H. Gupta N. Gill G. K. Choudhary A. (2024). Assesment of foliar micronutrient fertilization on leaf and crop growth attributes in sweet corn (Zea Mays L. saccharata). Commun. Soil Sci. Plant Anal. 55, 1–22. doi: 10.1080/00103624.2023.2285959

[B27] Lamlom S. F. Abdelghany A. M. Ren H. Ali H. M. Usman M. Shaghaleh H. . (2024). Revitalizing maize growth and yield in water-limited environments through silicon and zinc foliar applications. Heliyon 10, e35118. doi: 10.1016/j.heliyon.2024.e35118, PMID: 39157312 PMC11328083

[B28] Li J. Feng N. Zheng D. Du X. Wu J. Wang X. (2024). Regulation of seed soaking with indole-3-butyric acid potassium salt (IBA-K) on rapeseed (Brassica napus L.) seedlings under NaCl stress. BMC Plant Biol. 24, 904. doi: 10.1186/s12870-024-05586-4, PMID: 39350007 PMC11440911

[B29] Li J. Gao S. Bao C. Yan S. Ma C. Yan C. M. (2025). Mechanisms by which soil solution Fe2+ affects seedling growth of rice under rice straw return. Agronomy 15, 271. doi: 10.3390/agronomy15020271

[B30] Li Y. He Z. Ding Y. Wang S. (2020a). Recovery effect of root soaking in brassinosteroid (BR) on transplanting shock of hydroponically grown long-mat rice seedlings under mechanical transplanting. Chin. J. Of Rice Sci. 34, 539. doi: 10.16819/j.1001-7216.2020.0308

[B31] Li S. Jiang H. Wang J. Wang Y. Pan S. Tian H. . (2019). Responses of plant growth, physiological, gas exchange parameters of super and non-super rice to rhizosphere temperature at the tillering stage. Sci. Rep. 9, 10618. doi: 10.1038/s41598-019-47031-9, PMID: 31337786 PMC6650488

[B32] Li Y. Liang L. Fu X. Gao Z. Liu H. Tan J. . (2020b). Light and water treatment during the early grain filling stage regulates yield and aroma formation in aromatic rice. Sci. Rep. 10, 14830. doi: 10.1038/s41598-020-71944-5, PMID: 32908195 PMC7481283

[B33] Li X. Zhong Q. Li Y. Li G. Ding Y. Wang S. . (2016). Triacontanol reduces transplanting shock in machine-transplanted rice by improving the growth and antioxidant systems. Front. Plant Sci. 7. doi: 10.3389/fpls.2016.00872, PMID: 27379149 PMC4911394

[B34] Liang J. Kong L. Hu X. Fu C. Bai S. (2023). Chromosomal-level genome assembly of the high-quality Xian/Indica rice (Oryza sativa L.) Xiangyaxiangzhan. BMC Plant Biol. 23, 94. doi: 10.1186/s12870-023-04114-0, PMID: 36782126 PMC9926808

[B35] Lin T. Chen X. Ren Y. Qing B. Zhang M. Mo Z. . (2024). Effects of iron oxide nanocoatings on the seed germination, seedling growth, and antioxidant response of aromatic rice grown in the presence of different concentrations of rice straw extracts. J. Nanoparticle Res. 26, 78. doi: 10.1007/s11051-024-05986-5

[B36] Lin X. Fu Y. Li L. Zhao J. Wu L. Li Y. (2018). Effects of melatonin seed soaking on the recovery of rice variety ningjing 8 from mechanical injury during machine transplanting. Modern Agric. Sci. Technol. 19, 9–11, 13. doi: 10.3969/j.issn.1007-5739.2018.19.005

[B37] Lin L. Ma Q. Wang J. Lv X. Liao M. Xia H. . (2018). Effects of indole-3-butytric acid (IBA) on growth and cadmium accumulation in the accumulator plant Stellaria media. Environ. Prog. Sustain Energy 37, 733–737. doi: 10.1002/ep.12746

[B38] Liu X. Huang Z. Li Y. Xie W. Li W. Tang X. . (2020). Selenium-silicon (Se-Si) induced modulations in physio-biochemical responses, grain yield, quality, aroma formation and lodging in fragrant rice. Ecotoxicol Environ. Saf. 196, 110525. doi: 10.1016/j.ecoenv.2020.110525, PMID: 32224370

[B39] Liu Y. Ma J. Li F. Zeng X. Wu Z. Huang Y. . (2024). High concentrations of se inhibited the growth of rice seedlings. Plants 13, 1580. doi: 10.3390/plants13111580, PMID: 38891388 PMC11174541

[B40] Liu Q. Wu X. Ma J. Chen B. Xin C. (2015). Effects of delaying transplanting on agronomic traits and grain yield of rice under mechanical transplantation pattern. PloS One 10, e0123330. doi: 10.1371/journal.pone.0123330, PMID: 25875607 PMC4395310

[B41] Matusmoto T. Yamada K. Yoshizawa Y. Oh K. (2016). Comparison of Effect of brassinosteroid and Gibberellin biosynthesis inhibitors on growth of rice seedlings. Rice Sci. 23, 51–55. doi: 10.1016/j.rsci.2016.01.006

[B42] Min S. P.Paudel K. Feng C. (2021). Mechanization and efficiency in rice production in China. J. Integr. Agric. 20, 1996–2008. doi: 10.1016/S2095-3119(20)63439-6

[B43] Mohamed ASE-Y. Faisal M. A. M. (2021). Ameliorative effect of GA3 as foliar spray treatment on performances of salt-stressed damsisa (Ambrosia maritima L.). Plants 732.2, 58–70. doi: 10.22004/ag.econ.340590

[B44] Noreen S. Sultan M. Akhter M. S. Shah K. H. Ummara U. Manzoor H. . (2021). Foliar fertigation of ascorbic acid and zinc improves growth, antioxidant enzyme activity and harvest index in barley (Hordeum vulgare L.) grown under salt stress. Plant Physiol. Biochem. 158, 244–254. doi: 10.1016/j.plaphy.2020.11.007, PMID: 33221118

[B45] Pan S. Rasul F. Li W. Tian H. Mo Z. Duan M. . (2013). Roles of plant growth regulators on yield, grain qualities and antioxidant enzyme activities in super hybrid rice (Oryza sativa L.). Rice 6, 9. doi: 10.1186/1939-8433-6-9, PMID: 24280625 PMC4883720

[B46] Paque S. Weijers D. (2016). Q&A: Auxin: the plant molecule that influences almost anything. BMC Biol. 14, 67. doi: 10.1186/s12915-016-0291-0, PMID: 27510039 PMC4980777

[B47] Prom-u-thai C. Rerkasem B. Yazici A. Cakmak I. (2012). Zinc priming promotes seed germination and seedling vigor of rice. J. Plant Soil Sci. 175, 482–488. doi: 10.1002/jpln.201100332

[B48] Qing B. Jiang Y. Chen Y. Chen J. Xie H. Mo Z. (2022). Nitrogen modulates early growth and physio-biochemical attributes in fragrant rice grown under cadmium and multiwall carbon nanotubes stresses. Environ. Sci. PollutIion Res. 29, 67837–67855. doi: 10.1007/s11356-022-20432-6, PMID: 35524851

[B49] Ros C. Bell R. W. White P. F. (2003). Seedling vigour and the early growth of transplanted rice (Oryza sativa). Plant Soil 252, 325–337. doi: 10.1023/A:1024736104668

[B50] Santos M. Ferreira H. Aires A. Oliveira I. Sousa J. R. Raimundo F. . (2025). Enhancing sweet cherry quality: magnesium and potassium applications increase bioactive compounds and antioxidant activity. J. Sci. Food Agric. 105, 5840–5850. doi: 10.1002/jsfa.14294, PMID: 40243129

[B51] Shahzad K. Hussain S. Arfan M. Hussain S. Waraich E. A. Zamir S. . (2021). Exogenously applied Gibberellic Acid enhances growth and salinity stress tolerance of Maize through modulating the morpho-physiological, biochemical and molecular attributes. Biomolecules 11, 1005. doi: 10.3390/biom11071005, PMID: 34356629 PMC8301807

[B52] Singh H. Singh K. N. (2014). Transplanting shock in temperate rice and its influence on rooting characteristics and grain yield. Indian J. Agric. Res. 48, 389–393. doi: 10.5958/0976-058X.2014.01320.1

[B53] Singh Patel P. Kumar Singh S. Patra A. Jatav S. S. (2022). Root dipping, foliar and soil application of Zinc increase growth, yields, and grain Zinc in rice (Oryza sativa L.) grown in moderate Zinc soil of inceptisol order. Commun. Soil Sci. Plant Analysis. 53, 15. doi: 10.1080/00103624.2022.2069800

[B54] Stuepp C. A. Wendling I. Koehler H. S. Zuffellato-Ribas K. C. (2015). Rooting cuttings from epicormic shoots of Paulownia fortunei var. Mikado adult trees. Rev. Ciec. Florestal. 25, 667–677. doi: 10.5902/1980509819617

[B55] Subudhi P. K. (2023). Molecular research in rice. Int. J. Mol. Sci. 24, 10063. doi: 10.3390/ijms241210063, PMID: 37373210 PMC10298954

[B56] Takahashi N. Sunohara Y. Fujiwara M. Matsumoto H. (2017). Improved tolerance to transplanting injury and chilling stress in rice seedlings treated with orysastrobin. Plant Physiol. Biochem. 113, 161–167. doi: 10.1016/j.plaphy.2017.02.004, PMID: 28214729

[B57] Tao L. Ju G. Ling B. Yanan W. Jing H. Mahendar K. . (2019). Influence of green manure and rice straw management on soil organic carbon, enzyme activities, and rice yield in red paddy soil. Soil Tillage Res. 195, 104428. doi: 10.1016/j.still.2019.104428

[B58] Tuiwong P. Lordkaew S. Prom-u-thai C. (2021). Improving grain zinc concentration in wetland and upland rice varieties grown under waterlogged and well-drained soils by applying zinc fertilizer. Agronomy 11, 554. doi: 10.3390/agronomy11030554

[B59] Wahyuni S. Sinniah U. R. Yusop M. K. Amarthalingam R. (2016). Improvement of seedling establishment of wet seeded rice using GA3 and IBA as seed treatment. Indones J. Agric. Sci. 4, 56–62. doi: 10.21082/ijas.v4n2.2003.p56-62

[B60] Wu C. Dun Y. Zhang Z. Li M. Wu G. (2020). Foliar application of selenium and zinc to alleviate wheat (Triticum aestivum L.) cadmium toxicity and uptake from cadmium-contaminated soil. Ecotoxicol Environ. Saf. 190, 110091. doi: 10.1016/j.ecoenv.2019.110091, PMID: 31881404

[B61] Xing Z. Hu Y. Qian H. Cao W. Guo B. Wei H. . (2017). Comparison of yield traits in rice among three mechanized planting methods in a rice-wheat rotation system. J. Integr. Agric. 16, 1451–1466. doi: 10.1016/S2095-3119(16)61562-9

[B62] Ye D. Yu Z. Chen J. Wei S. Zhang Z. Huang S. . (2025). Foliar magnesium application improves sweet corn yield: boosting nutrient uptake and grain carbohydrate under dense planting condition. Front. Plant Sci. 16, 1499391. doi: 10.3389/fpls.2025.1499391, PMID: 40416090 PMC12098614

[B63] Yu L. Hong H. Yong A. Shao H. Zheng H. She T. . (2019). Enhancing grain yield and operational feasibility by transplanting young hydroponically grown long-mat rice seedlings. Agron. J. 111, 2303–2313. doi: 10.2134/agronj2018.09.0571

[B64] Zhang H. Liu X.-L. Zhang R.-X. Yuan H.-Y. Wang M.-M. Yang H.-Y. . (2017). Root damage under alkaline stress is associated with reactive oxygen species accumulation in rice (Oryza sativa L.). Front. Plant Sci. 8, 1580. doi: 10.3389/fpls.2017.01580, PMID: 28943882 PMC5596797

[B65] Zhu G. Ren Z. Liu Y. Lu F. Gu L. Shi Y. . (2020). Optimization of leaf properties and plant phenotype through yield-based genetic improvement of rice over a period of seventy years in the Yangtze River Basin of China. Food Energy Secur 9, e223. doi: 10.1002/fes3.223

[B66] Zhuang J. Fang Y. Zheng J. Duan Y. Liu X. Mo Z. (2025). Effects of selenium foliar spraying on seedling growth and stem sheath hardness in fragrant rice. Agriculture 15, 335. doi: 10.3390/agriculture15030335

